# Cervid Prion Protein Polymorphisms: Role in Chronic Wasting Disease Pathogenesis

**DOI:** 10.3390/ijms22052271

**Published:** 2021-02-25

**Authors:** Maria Immaculata Arifin, Samia Hannaoui, Sheng Chun Chang, Simrika Thapa, Hermann M. Schatzl, Sabine Gilch

**Affiliations:** 1 Department of Comparative Biology & Experimental Medicine, Faculty of Veterinary Medicine, University of Calgary, Calgary, AB T2N 4N1, Canada; maria.arifin@ucalgary.ca (M.I.A.); shannaou@ucalgary.ca (S.H.); shengchun.chang@ucalgary.ca (S.C.C.); sthapa@ucalgary.ca (S.T.); hschaetz@ucalgary.ca (H.M.S.); 2 Calgary Prion Research Unit, University of Calgary, Calgary, AB T2N 4N1, Canada; 3 Hotchkiss Brain Institute, University of Calgary, Calgary, AB T2N 4N1, Canada

**Keywords:** chronic wasting disease, prion protein, cervid, polymorphism, strain, pathogenesis

## Abstract

Chronic wasting disease (CWD) is a prion disease found in both free-ranging and farmed cervids. Susceptibility of these animals to CWD is governed by various exogenous and endogenous factors. Past studies have demonstrated that polymorphisms within the prion protein (PrP) sequence itself affect an animal’s susceptibility to CWD. PrP polymorphisms can modulate CWD pathogenesis in two ways: the ability of the endogenous prion protein (PrP^C^) to convert into infectious prions (PrP^Sc^) or it can give rise to novel prion strains. In vivo studies in susceptible cervids, complemented by studies in transgenic mice expressing the corresponding cervid PrP sequence, show that each polymorphism has distinct effects on both PrP^C^ and PrP^Sc^. It is not entirely clear how these polymorphisms are responsible for these effects, but in vitro studies suggest they play a role in modifying PrP epitopes crucial for PrP^C^ to PrP^Sc^ conversion and determining PrP^C^ stability. PrP polymorphisms are unique to one or two cervid species and most confer a certain degree of reduced susceptibility to CWD. However, to date, there are no reports of polymorphic cervid PrP alleles providing absolute resistance to CWD. Studies on polymorphisms have focused on those found in CWD-endemic areas, with the hope that understanding the role of an animal’s genetics in CWD can help to predict, contain, or prevent transmission of CWD.

## 1. Introduction

Chronic wasting disease (CWD) is a prion disease, or transmissible spongiform encephalopathy (TSE), found in cervid species, such as elk, deer, reindeer, and moose [1]. It is an infectious and fatal neurodegenerative disease with no prophylaxis or cure available [1]. Prions are proteinaceous infectious particles consisting of PrP^Sc^, an abnormally folded and infectious isoform of the endogenous prion protein (PrP^C^). Prions can convert PrP^C^ into PrP^Sc^, leading to accumulation of aggregated PrP^Sc^ in the central nervous system (CNS) and ultimately death [2]. Pronounced weight loss is a hallmark in animals with CWD, thus the term ‘wasting disease’ [1]. Another important feature of CWD is that CWD prions, abbreviated as PrP^CWD^ here onwards, are very contagious [1]. This is because PrP^CWD^ disseminates throughout the body of the infected cervid. PrP^CWD^ has been detected in the lymphatic system, salivary gland, intestinal tract, muscles, and blood, as well as urine, saliva, and feces, of infected cervids [3,4,5,6,7,8,9,10,11,12,13,14]. PrP^CWD^ is released into the environment through bodily fluids and excreta and bind to soil and plants, remaining infectious even after decades [15,16,17,18,19]. Prolonged PrP^CWD^ shedding and its persistence in the environment leads to efficient lateral transmission between both farmed and free-ranging cervids [10,14,20,21,22,23].

**Figure 1:**
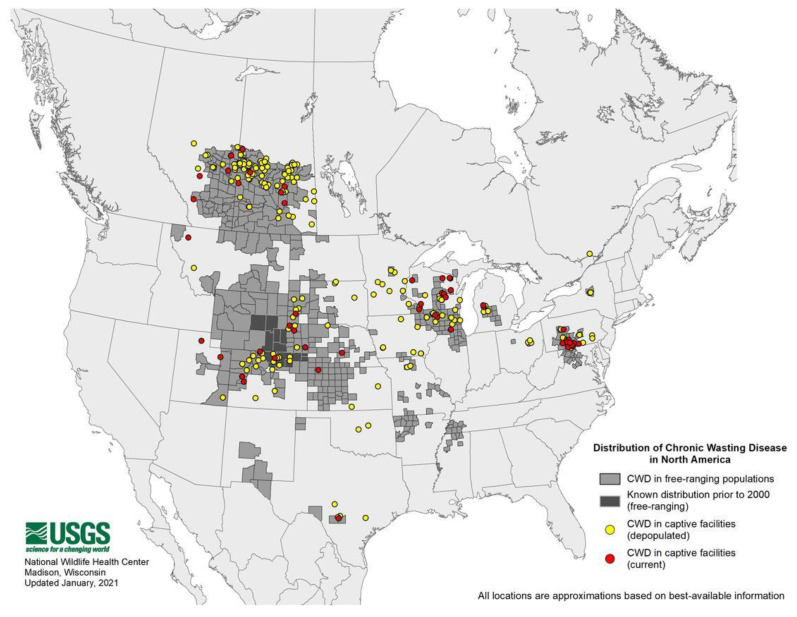
Chronic wasting disease (CWD) distribution in North America. Courtesy of the U.S. Geological Survey National Wildlife Health Center.

**Figure 2:**
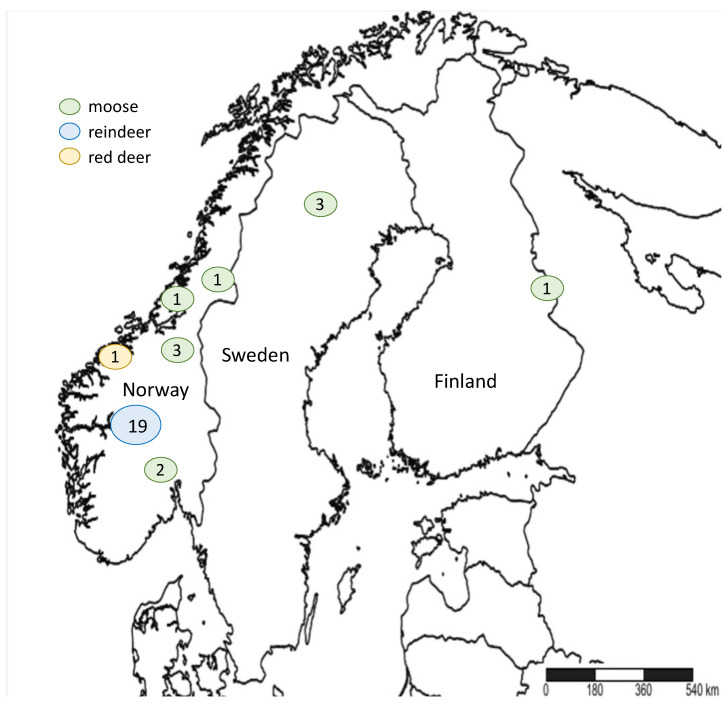
Chronic wasting disease (CWD) distribution in Scandinavia, based on Mysterud et al., 2020 [35]. Numbers in circles represent the number of CWD-positive animals.

CWD was first reported by the late E.S. Williams in captive mule deer in Colorado in the 1970s [24] and later on in free-ranging elk in 1981 [25]. In 1996, Saskatchewan reported the first case of CWD in Canada [26]. The disease was also reported in South Korea as a case of imported elk from Canada [27]. CWD is currently found in 26 U.S. states and 3 Canadian provinces (Figure 1) [28]. This efficient spread highlights the contagiousness and difficulty to contain CWD. Wild and farmed cervid species known to be naturally affected by CWD in North America include white-tailed deer (*Odocoileus virginianus*), mule deer (*O. hemionus*), elk (*Cervus canadensis*), red deer (*C. elaphus*), and moose (*Alces alces* sp.) [29]. In 2016, the first case of CWD in Europe was reported in free-ranging Norwegian reindeer (*Rangifer tarandus tarandus*), followed by reports in red deer and moose [30,31]. It was also recently reported in moose in Finland and Sweden (Figure 2) [32,33,34]. To date, the origin of CWD is not well known.

**Figure 3:**
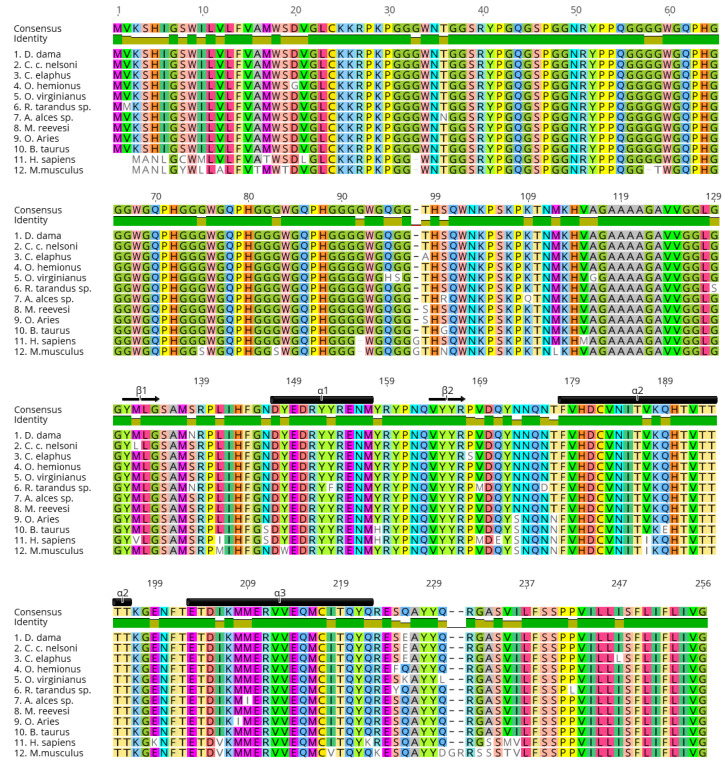
Cervid prion protein sequence alignment showing conserved homology between species. Protein alignment was performed in Geneious v10.2.6 (https://www.geneious.com) (accessed on 15 December 2020) using the ClustalW algorithm. Amino acid numbering is based on the consensus sequence. Amino acid variants were added manually to each sequence and are shown in white boxes. NCBI accession numbers used in this alignment: (1) QAU19527.1, (2) ABW79881.1, (3) QAU19537.1, (4) AAO91945.1, (5) QKI87491.1, (6) AAT77253.1, (7) QHZ32187.1, (8) AGU92564.1, (9) ABA08026.1, (10) BAI50003.1, (11) BCK59655.1, (12) CAJ18553.1. β1, β2: first, second beta-strand; α1, α2, α3: first, second and third alpha-helix (based on mouse PrP numbering [136]). Refer to Table 1 for cervid species names in sequences 1–8. Non-cervid species names for sequences 9–12: O. = Ovis, B. = Bos, H. = Homo and M. = Mus.

While CWD is efficiently transmitted among and between cervid species, prions in general do not easily transmit from their main host species to other species due to the presence of transmission barriers [36,37,38,39,40,41,42]. The host’s PrP primary sequence homology with the incoming PrP^Sc^ is an important factor in the PrP^C^ to PrP^Sc^ conversion [38,43,44,45]. Mismatch between the substrate (PrP^C^) and template (PrP^Sc^) can result in less efficient conversion and hinder disease transmission [46,47,48]. Furthermore, the replication environment and presence of cofactors also play a role in successful prion propagation [49,50,51,52,53,54,55,56]. Studies show that different prion conformers or strains can propagate within a single host, resulting in a prion ‘cloud’ or isolate, containing a mixture of strains [57,58,59,60]. The prion strain concept is highly debated, but the generally accepted notion is that when a prion inoculum retains its disease phenotype in vivo and biochemical features in vitro through serial passages, it is recognized as a distinct strain [41,61,62,63,64,65,66,67]. However, prions can also ‘jump’ from one species to another, including zoonotic transmission. An important example of this phenomenon is bovine spongiform encephalopathy (BSE) that was transmitted to humans in the form of variant Creutzfeldt-Jakob Disease (vCJD) [68,69,70], as well as to a number of other species, e.g., exotic and domestic cats or exotic ungulates [71,72,73,74,75,76,77]. The molecular mechanisms of how prions adapt to a new host are not well understood. One possible mechanism is the selection of prion strain(s) preferred by the host PrP^C^, enabling prion propagation [78,79]. Another is that the incoming PrP^Sc^ can adapt to the host’s PrP^C^ conformation(s), or vice versa, sometimes resulting in the emergence of a new prion strain [78,80,81]. A 2019 study by Beringue and colleagues suggested that cross-species transmission can occur due to the cooperation between sub-assemblies of prion conformations [82,83]. One known molecular determinant or ‘switch’ of the CWD transmission barrier in this regard is the PrP β2-α2 loop [84,85,86,87,88,89,90,91]. Replacing the β2-α2 loop sequence of different species with that of cervids resulted in increased misfolding of PrP and the transmissibility of CWD to these species [89,90,91]. Furthermore, it has been demonstrated that the prion transmission barrier can be modulated by non-synonymous single nucleotide polymorphisms (SNPs) within the prion protein gene (*Prnp*) [57,92,93]. In fact, selecting for prion-resistant *Prnp* alleles in breeding programs has been shown, or been predicted, to curb the spread of scrapie in sheep and goats [94,95,96,97]. Although the PrP sequence is highly conserved among cervid species (Figure 3), it has some variations [98,99]. Studies show that these polymorphisms contribute in modulating cervid susceptibility to CWD (Table 1) and PrP^CWD^ strain propagation [93,99,100,101].

## 2. *Prnp* Polymorphisms in Cervids

The *Prnp* coding sequence is extremely conserved among cervid species (Figure 3); however, there are numerous key polymorphisms that have been identified and, for some, well-characterized that they are associated with lower rates and/or delayed CWD disease progression (Table 1). Very early on, even when CWD was not as widespread as today, genetic analyses suggested the existence of a polymorphism at codon 132 in the North American elk PrP sequence that encodes either methionine (M) or leucine (L), with the 132M being the more frequent (wild-type) allele [112]. This report was of interest because codon 132 in elk is equivalent to codon 129 in the human *PRNP* which encodes either M or valine (V). Codon 129 in the human *PRNP* has been described as a key polymorphism influencing susceptibility to prion diseases [137,138], and more particularly, to the BSE agent. Shortly after, O’Rourke and colleagues confirmed the existence of this dimorphism in elk [139], and they reported that 132M homozygotes were over-represented in free-ranging, as well as farmed elk infected with CWD, when compared to healthy animals. Based on that, they suggested, for the first time, the existence of a relative protection of animals carrying at least one allele encoding L at codon 132 (132LL and 132ML) against CWD [113]. This suggestion was later conflicted by a study of Perucchini and colleagues who instead showed that in a survey of free-ranging elk in Colorado, each genotype was represented in CWD-positive animals in proportion to their frequency in the population [116]. However, the protective effects against CWD-infection of 132L have been validated in an experimental setting [114,115]. To compare the genetic susceptibility of elk, Hamir and colleagues have orally challenged elk with 132MM, 132ML, or 132LL genotypes with brain material from a pool of 132MM and 132ML elk infected with CWD [114]. The results of this study suggested that 132LL elk may have reduced susceptibility to oral infection with CWD as they did not develop disease up to the point when 132MM and 132ML animals developed clinical signs of disease, though there was a significant difference in incubation time between the latter two, as well [114]. Shortly after, the same group reported that 132LL animals were indeed susceptible to CWD infection but with incubation periods approximately 1.5 times longer than 132ML elk and 3 times longer than those homozygous for 132M [115]. Compared to 132MM, disease in 132LL elk was characterized by differences in spongiform changes, PrP^CWD^ distribution and accumulation, and higher PrP^CWD^ fibril stability, which led to a significant negative correlation between relative amount of PrP^CWD^ and incubation periods [115]. A recent study from the same group confirmed that these characteristics were retained in passages in transgenic mice expressing 132L elk PrP [140].

Comparisons of the *Prnp* coding sequence of mule deer and Rocky Mountain elk revealed that these two species have identical PrP sequences, with the exception of codon 226 that encodes glutamic acid (E) in elk and glutamine (Q) in mule deer [119]. Extensive genetic analyses revealed the presence of a polymorphism at codon 20 of the mule deer sequence, which encodes either aspartate (D) or glycine (G), and at position 225, encoding serine (S) or phenylalanine (F), with a frequency of 0.85 of the dominant alleles (D20 and S225) [141]. Jewell and colleagues showed that the infection rate of CWD was 30 times higher in mule deer homozygous for S at position 225 compared to heterozygous animals (225SF) in the CWD endemic areas of Wyoming and Colorado [110]. In fact, while the combined frequency of heterozygous 225SF and homozygous 225FF mule deer was 9.3%, these animals represent only 0.3% of the sampled population that developed CWD [110]. Another study conducted on 19 mule deer orally challenged with CWD and followed from 3 to 26 months after inoculation showed that peripheral and central PrP^CWD^ accumulation and deposition were comparable between 225SS and 225SF mule deer at the terminal stage of disease (19 to 23 and 36 months post-infection, respectively). However, the time course of PrP^CWD^ distribution was significantly different between the different genotypes (SS vs. SF). In 225SF animals, deposition of PrP^CWD^ in lymphatic tissues and in the CNS was significantly delayed compared to 225SS animals. In fact, 225SS mule deer developed spongiform lesions after approximately 19 months post-infection, while 225SF animals were still asymptomatic, without any neuropathological lesions for up to 25 months post-infection [111]. Later studies showed that CWD susceptibility in mule deer with different *Prnp* genotypes at position 225 was also different. Though all animals (SS and FF) became infected with CWD, clinical disease manifestations were more subtle and detection of PrP^CWD^ with standard methods was inconclusive in 225FF mule deer, with presence of spongiform encephalopathy and the absence of detectable PrP^CWD^ deposits in the brain [142]. Apart from this, CWD in 225FF animals, in general, presented a more subtle, atypical trait and negative PrP^CWD^ immunoreactivity in the lymph nodes and obex [142]. Subsequent studies in transgenic mice expressing 225F-mule deer PrP^C^ further confirmed the effects of this polymorphism on CWD susceptibility [42].

The presence of a universally processed, but unexpressed, pseudogene (*Prnp*ψ) encoding asparagine (N) at codon 138 was initially reported in mule deer [141]. This complicated the analysis of genetic susceptibility of mule deer to CWD infection because the primers used failed to discriminate between the functional *Prnp* from *Prnp*ψ. O’Rourke and colleagues confirmed the presence of the *Prnp*ψ pseudogene in a study conducted in Nebraska of captive white-tailed deer [106]. They identified two alleles in the pseudogene encoding five or six copies of the octapeptide repeat, both of which encode N at codon 138 [106]. Later on, it was shown that the polymorphism at codon 138 was not unique to the *Prnp*ψ pseudogene but was also a feature of the functional *Prnp* gene in certain cervid species. Fallow deer are all homozygous for N at codon 138 [122]. Reindeer/caribou are found to also carry the S138N polymorphism, but not mule deer and white-tailed deer [122,123,131]. The 138N allele is present in caribou herds in North America in frequencies between 0.2–0.64 depending on population and subspecies, but it has not been reported in wild reindeer in Norway [130,132,133,135]. Interestingly, a significantly higher frequency of the 138N allele was detected in barren-ground compared to woodland caribou herds, with the exception of the Chinchaga woodland population [132,133]. The presence of the 138N allele was shown to be associated with reduced susceptibility to CWD upon natural routes of infection [123,124,131,134]. Fallow deer, which are homozygous for N at position 138, were resistant to natural CWD infection, suggesting that the presence of the 138N allele confers a relative protection, or, at least, delays the progression of the disease in this species. However, considering that intracerebral (i.c.) inoculation resulted in CWD infection, albeit with prolonged survival times [123], this shows that 138N PrP^C^ can be converted to PrP^CWD^ in vivo and, thus, does not confer an absolute protection against CWD. In parallel, Mitchell and colleagues also showed that the polymorphism at position 138 influences reindeer susceptibility to CWD upon oral infection [131]. They first suggested that homozygosity for S at codon 138 of the reindeer *Prnp* gene was associated with susceptibility to the CWD agent, while the presence of one 138N allele conferred resistance against it [131]. Later on, it was shown in another study that reindeer carrying the N allele eventually developed disease upon natural CWD infection [134]. However, PrP^CWD^ distribution in 138SN or NN animals was mostly limited to lymphoid tissues with significantly lower PrP^CWD^ burden compared to 138SS animals [134]. To further prove this, our lab has generated and inoculated gene-targeted mice expressing wt and 138N-cervid PrP through i.c. and intraperitoneal (i.p.) routes with various PrP^CWD^ isolates. 138NN mice did not develop clinical disease (up to ±660 dpi), while their 138SS (wt) PrP counterparts succumbed to disease at ±450 dpi upon i.c. inoculation, with confirmatory protease resistant PrP (PrP^res^) on immunoblot (unpublished data).

O’Rourke and colleagues confirmed previous studies done in smaller cohorts [39,102,143] by determining the *Prnp* genotypes and CWD status in a group of 113 captive white-tailed deer (WTD) in west Nebraska [106]. In this study, half of the WTD tested for PrP^CWD^ were positive in the brainstem or lymphoid tissues and three SNPs in *Prnp* were identified, at position 95 with alleles encoding either glutamine (Q) or histidine (H), at position 96 encoding either G or S and at position 116 encoding alanine (A) or G. A study by Johnson and colleagues in Wisconsin free-ranging WTD comparing the *Prnp* genotypes of CWD-positive and -negative WTD to determine the impact of gene modulation on CWD susceptibility revealed a polymorphism in the WTD *Prnp* gene at position 226 encoding either Q or lysine (K) [103]. Most importantly, by comparing the allelic frequencies of CWD-affected and CWD-negative WTD, they suggested that the presence of H and S at codon 95 and 96, respectively, was associated with reduced susceptibility to CWD [103]. However, although the presence of the 96S allele among CWD-infected deer was associated with slower disease progression and decreased PrP^CWD^ deposition compared to animals homozygous for 96G, the 96S animals were not completely resistant to the CWD agent as a CWD-affected homozygous 96S WTD was identified in this study [103]. Others reported a reduced CWD prevalence linked to the presence of the 96S allele [108,144] and that WTD homozygous for 96G had a four times greater risk of CWD infection [144]. Although the occurrence of the 95H allele is very rare (1–2%) in wild WTD population, the fact that animals carrying this allele are affected by CWD at an even lower rate in proportion to its allelic frequency suggests that it confers a relative protection against CWD [101,103,106,109,145]. In an experimental study, WTD with different *Prnp* alleles, wt (95Q/96G), 96S/wt (96S/95Q), 95H/wt (95H/96G), or 95H/96S were inoculated orally with PrP^CWD^ originating from wt (95Q/96G) WTD, to test the effect of the *Prnp* polymorphism on CWD susceptibility [105]. While all inoculated WTD succumbed to clinical CWD, a remarkable difference was observed in average survival periods between inoculated WTD harboring wt and other *Prnp* genotypes [105]. Wild type CWD-affected WTD had an average survival of less than 2 years, while 96S/wt CWD-affected WTD succumbed 9 months later [105]. Interestingly, WTD carrying the 95H allele showed the longest survival for up to 2.5 years post-infection [105]. Further analysis showed altered peripheral prion distribution in the 95H animals [105]. Changes in the biological and biophysical properties demonstrated the emergence of a new PrP^CWD^ strain, later referred to as 95H^+^ [93,104,146,147]. The effects of these polymorphisms were faithfully reproduced in wt 96G or 96S transgenic mice [42,148,149].

Another polymorphism with a very low frequency in the wild population is the A116G polymorphism in WTD. Although its influence on CWD susceptibility is unclear, with either no effect [106] or potential reduced susceptibility [92], this polymorphism is of certain interest because of its position in the highly conserved central hydrophobic core (HC) domain of PrP that is involved in prion conversion [150,151,152,153]. It is equivalent to codon 113 in human *PRNP*, located in the HC domain where other mutations are responsible for heritable prion disease [154].

In North American moose (*Alces alces* spp), polymorphisms at position 36, encoding threonine (T) or N, at position 100 encoding either S or arginine (R), and at position 209 encoding either M or isoleucine (I) have been reported [98,127,129]. The PrP sequence of European moose (*A. a. alces*) has high homology to the North American moose with the exception of the variant K to Q at position 109 [98]. All CWD cases identified to date were in moose carrying the wild type PrP sequence [32]. However, CWD-positive cases in moose are rare compared to other cervids; therefore, it is not possible to draw conclusions about how these polymorphisms could modulate CWD susceptibility.

## 3. Cervid PrP Polymorphisms and Effects on PrP^C^ Structure

There is strong evidence demonstrating that cervid *Prnp* polymorphisms can affect susceptibility to CWD, raising the question how these single amino acid substitutions affect PrP^C^ folding. Studies utilizing in silico molecular dynamics (MD) simulation, as well as high-resolution structure data obtained from nuclear magnetic resonance (NMR) spectroscopy, have provided valuable biophysical information; however, only a very limited number of the known polymorphisms were analyzed.

An interesting feature of the cervid PrP structure is that the loop between the second β-sheet (β2) and second α-helix (α2) is extremely well defined, which makes it more rigid compared to other species, due to amino acid substitutions from S to N at position 170, and N to threonine (T) at position 174 (Figure 3; mouse PrP numbering) [84,90]. Overexpression of the S170N/N174T or ‘rigid loop’ PrP in transgenic mice has been shown to induce spontaneous de novo prion disease [84]. Furthermore, this structure also plays a key role in PrP^CWD^ transmissibility to different species, including to humans [85,87,155,156].

The distal region of the third α-helix (α3) is known to interact with the β2-α2 loop, resulting in a protein surface epitope that affects the conversion of PrP^C^ to PrP^Sc^ [42,88,157]. Interestingly, when the β2-α2 loop of mouse PrP is replaced with the S170N/N174T rigid loop, the α3 helix up to codon 226 also becomes more well defined [88]. Recent studies using MD simulations to explore the effects of substituting amino acids at codons 225 and 226 in cervid PrP show that a F and Q at these respective positions, which represents the 225F PrP allele found in mule deer less susceptible to CWD infection, allows for the formation of side chain hydrogen bonds between the tyrosine (Y) at codon 228 of the α3 helix and the aspartate (D) at codon 170 of the β2-α2 loop, thus likely resulting in a more stable structure [42].

MD simulations on wt 116A and 116G WTD PrP revealed that the latter has a less stable conformation, based on several parameters. The 116G PrP conformer, when compared to the wt conformer, has greater structural fluctuations and is less compact with a larger radius of gyration [92]. The 116G conformer also has a greater propensity to form β-strands, where in the rigid loop and portions of the hydrophobic domain it has a higher proportion of β-strand secondary structure [92]. In addition, this polymorphic variant has a slightly larger solvent accessible surface area and hydrophobic residue exposure, indicating a weaker solvent-residue interaction, thus predisposing the structure to self-assembly [92]. These in silico findings are corroborated by real-time quaking induced conversion assays (RT-QuIC), a sensitive cell-free prion amplification technique involving the successive cycles of shaking and incubating the prion seed in a mixture of recombinant PrP (rPrP) and Thioflavin T that fluoresces upon binding to amyloid aggregates [158]. When using 116G or 116A rPrP substrates and CWD-infected brain homogenate from WTD as a seed, 116G rPrP exhibited a higher conversion rate [92,159]. Regarding elk *Prnp* polymorphisms, the 132L substrate had lower amplification rates and extension efficiency than that of 132M and wild-type deer substrates [159]. However, it needs to be determined whether this accelerated conversion in vitro translates into an in vivo situation. Similar MD analyses was performed on the 96S and 95H WTD PrP polymorphisms, both associated with reduced susceptibility to CWD infection. These studies indicated that 96S PrP is less stable than wt and 95H conformers, which were similar [160].

Overall, studies investigating structural effects and the impact of polymorphisms on aggregation and conversion propensity are very limited. More work is needed to identify correlations between PrP stability and CWD susceptibility.

## 4. Prion Protein Polymorphisms and PrP^CWD^ Strains

As previously mentioned, prions exist as different conformers, to which we refer as strains. The strain phenomena can occur spontaneously, or due to mutation and selection during the process of adaptation in a new host [57]. The presence of prion strains was first reported in goats [161,162] and later on in other species, including cervids [163]. A prion strain is considered a distinct strain when they present consistent biological and biochemical characteristics, e.g., stability in denaturing agents, distribution in the CNS, tropism in different tissues, incubation period in vivo, and retain these features upon serial passage in animal models [41,64,66,67,164].

Angers and colleagues reported the first presence of PrP^CWD^ strains in both deer and elk, identified as CWD1 and CWD2 [163]. First and second passages of these isolates into transgenic mice overexpressing deer PrP (Tg(CerPrP)1536) show that each strain retained its distinct characteristics, i.e., differences in disease incubation times and neuropathological profiles [163]. However, elk seemed to propagate more distinctly CWD1 or CWD2 strains compared to a more ‘mixed’ phenotype in deer [163]. Transmission into transgenic mice expressing elk PrP [165] indicated that expressing the 226E allele results in more stable strains, whereas the 226Q deer PrP generates unstable strains resulting in less distinguishable phenotypes with subsequent passages. This indicates that the host’s PrP plays a role in determining how the strains are being propagated in these transgenic mice [163]. The effect of these residues was later confirmed in gene-targeted mice expressing deer (GtQ226) or elk (GtE226) PrP [120].

Protein conformational stability, which is determined by denaturation with guanidium hydrochloride, can be used to distinguish prion variants [166]. This readout revealed that the 226Q deer PrP^CWD^ has a greater conformational stability than that of the elk (226E), demonstrated in the GtE226 and GtQ226 mice [120], as well as CWD-infected RK13 cells [167]. In mule deer PrP, where 225F is known to be the less susceptible allele, studies suggest the possibility of a different strain [42]. PrP^Sc^ distribution patterns in tgDeerPrP-F225 mice were altered compared to inoculated tgDeer mice. TgDeerPrP-F225 mice showed diffuse and more widespread PrP^CWD^ deposition in the thalamus but not the corpus callosum, while inoculated tgDeer (wt) mice were characterized by continuous, symmetrical plaque deposits throughout the hippocampal alveus [42].

The impact of the elk M132L polymorphism was assessed in in vitro and in vivo studies. Using 132L rPrP as a substrate in RT-QuIC resulted in longer lag times (time to reach the positivity threshold) compared to 132M substrate seeded with PrP^CWD^ from 132MM or ML animals [118]. These in vitro data, together with the in vivo studies in both elk and mice mentioned above [115,118], indicated that CWD-infected elk with various genotypes may carry distinct prion strains. Strain typing experiments have been carried out using transgenic mouse models expressing M132 or L132 elk prion protein [140], strengthening the argument that this *Prnp* polymorphism modulates the isolation of a novel PrP^CWD^ strain.

Transgenic mice expressing wt-deer PrP^C^ (tg33) or 96S PrP^C^ (tg60) were generated to assess the effects of the polymorphism at position 96 [148]. Tg33 mice developed disease as early as 160 dpi when challenged with PrP^CWD^, with typical vacuolation and deposition of PrP^CWD^ in the brain [148]. Some animals showed extensive neuronal loss and apoptosis in the hippocampus and cerebellum, and extraneuronal PrP^CWD^ accumulation was found in the spleen and intestinal tissue [148]. In contrast, inoculated tg60 mice did not show any evidence of prion disease nor propagation of PrP^CWD^ at over 600 dpi [148]. When the same lines were inoculated with CWD-affected 96SS deer brain homogenate, tg33 mice developed clinical signs of disease and succumbed to the 96SS prions, while tg60 inoculated mice were not susceptible to the same inoculum [149]. Interestingly, tg60 mice inoculated with brain homogenate from inoculated tg33 mice showed no transmission suggesting that 96S PrP played an inhibitory role in disease progression in these mice [149]. In addition, heterozygous mice were generated by breeding homozygous 96G and 96S mice and challenged with PrP^CWD^ [149]. Heterozygosity resulted in delayed disease in these mice, once again showing that, although it does not provide complete resistance, this allele plays an important role in CWD susceptibility [149]. In fact, transgenic mice, tg(DeerPrP-S96), overexpressing 96S-deer PrP, and tg(DeerPrP-H95) were generated and challenged with different prion agents [42]. As expected, substitutions at residues 95 and 96 inhibited PrP^CWD^ propagation [42]. All challenged tg(H95) animals remained free of clinical signs, while tg(S96) animals showed an incomplete attack rate with delayed incubation times [42]. It, therefore, appears that substitutions in the unstructured region of PrP, at residues 95 and 96 affect prion conversion in a strain/species-specific manner. However, in Angers’ study [42], tg(S96) mice were only partially resistant to PrP^CWD^ and this somewhat conflicts the tg60 studies that showed a complete resistance to PrP^CWD^ [148,149]. Furthermore, deer homozygous for 96S were found to be susceptible to CWD [108], which corroborates Anger’s findings. For this reason, the complete resistance of tg60 mice to PrP^CWD^ was imputed to the low transgene expression in these mice (70% of physiological expression), but it was later demonstrated otherwise [93]. Tg60 mice succumbed to prion disease only when inoculated with PrP^CWD^ from deer expressing the 95H allele (95H/95Q and 95H/96S). Incubation periods in diseased tg60 mice decreased upon passages suggesting an adaptation of the 95H^+^ PrP^CWD^ agent. These series of experiments revealed that 95H^+^ was an emergent strain that adapted when passaged in mice expressing 96S-PrP, confirming once again that *Prnp* polymorphisms modulate the emergence and selection of novel strains [93]. In addition to transmissions in wt and 96S-PrP mice, 95H^+^ prions were also proven to be different following transmission into Syrian golden hamsters and C57BL/6 mice [146,147]. In fact, 95H^+^ only induced clinical disease in C57BL/6 but not in hamsters. Similarities between conformational stabilities of PrP^CWD^ from the brains of 95H^+^-tg60 mice and PrP^CWD^ from brains of 95H/96S deer revealed that the 95H^+^ strain was in fact a product of the replication of Wisc-1 (PrP^CWD^ originating from a wt WTD field isolate) by 95H-PrP^C^ [147].

We have characterized the impact of a polymorphism at codon 116 (A to G) of the WTD PrP on PrP^C^ and PrP^CWD^ properties and susceptibility [92]. We compared PrP^CWD^ obtained from a heterozygous 116AG hunter-harvested wild deer to that of the Wisc-1 wt WTD isolate. When tgDeer (tg(CerPrP)1536^+/+^) mice were inoculated with either wt 116AA (Wisc-1) or 116AG prions, mice showed significant differences in terms of disease progression and survival [92]. Wisc-1 inoculated mice had a rapid disease progression and short survival compared to 116AG-inoculated mice, which had a delayed disease progression by approximately 2 months and prolonged survival [92]. Upon subsequent passages, inoculated PrP^CWD^ adapted to their new host (tgDeer) with shortened survival, but disease progression and survival times were still significantly different between the two PrP^CWD^ isolates [92]. Furthermore, distinct biochemical features of the two PrP^CWD^ isolates were retained upon passage in tgDeer mice. Tg60 mice inoculated with the 116AG isolate succumbed to prion disease with a survival time and biochemical signature different from that of 95H^+^, the only PrP^CWD^ strain previously known to induce disease in tg60 mice (unpublished data). This finding strongly suggests that 95H^+^ and 116AG are distinct PrP^CWD^ strains. 116AG PrP^CWD^ derived from deer brain samples were also found to be conformationally less stable than the wild type 116AA PrP^CWD^ [92]. Primary cerebellar granular neuron (CGN) cultures generated from tgDeer mice [168] were found to facilitate improved propagation of 116AA over 116AG PrP^CWD^ [92]. RT-QuIC assay also showed that 116AG PrP^CWD^ have reduced seeding activity compared to 116AA PrP^CWD^; however, 116G rPrP forms amyloid fibrils in vitro more readily than 116AA rPrP [92]. All together, these results strongly support the existence of a new distinct strain, 116AG that is different from Wisc-1 and 95H^+^ PrP^CWD^ strains. Our findings suggest that the presence of the 116AG PrP^CWD^ may strongly impact the replication of the wt/Wisc1 PrP^Sc^. While it is still unclear how 116AG strains were generated, novel strains with such specific characteristics might impact the ability of PrP^CWD^ to cross species barriers among cervid and non-cervid species.

## 5. PrP^CWD^ Shedding in Cervid Species

PrP^CWD^ are highly contagious as these prions, either amplified in the CNS and anterogradely transported to the periphery, or amplified in these extraneural organs themselves, are shed through excrements and bodily fluids (feces, urine, saliva, blood, etc.) of infected cervids [3,8,11,12,13,14,169,170,171,172]. This facilitates direct transmission by animal contacts, as well as indirect transmission through contaminated soil and foliage, followed by transport to the CNS through peripheral nerves, typically those innervating organs of the digestive and lymphatic system [169,173,174,175,176,177]. In addition, transmission by blood transfusion and the placenta have been shown [178,179]. The infectious dose of PrP^CWD^ in saliva and urine of infected deer and transgenic mice was determined in several studies [6,10,180]. Saliva seemed to be the most infectious material in infected cervids, with PrP^CWD^ concentrations similar to 10^−6^ to 10^−8^ CWD-positive brain dilutions and 10-fold than that of urine [10,180]. The critical question arises whether prolonged shedding occurs due to polymorphisms affecting susceptibility to CWD. Although few studies have been done to investigate the effect of cervid PrP polymorphisms on PrP^CWD^ shedding, some indicate that differences do occur [8,10,14,99,101,111,181,182]. Plummer and colleagues suggested that less susceptible cervid *Prnp* genotypes, such as 132ML in elk, 225SF in MD and 96SS in WTD, shed less PrP^CWD^ in their urine and feces [181], in agreement with other studies [11,14]. However, other studies also investigating the G96S polymorphism reported little difference in PrP^CWD^ detection in saliva and urine of 96GS and SS animals [10], and in the blood of 96GG and GS animals [182]. However, interpretation of these results may be confounded by incompatibilities of the seed and substrate in their detection methods, i.e., RT-QuIC and Protein Misfolding Cyclic Amplification (PMCA), respectively. A study on mule deer PrP polymorphisms reported that 225SF mule deer were subclinical for longer periods of time but, nevertheless, had detectable PrP^CWD^ in their lymph nodes, suggesting possibilities of longer shedding periods [111]. These studies are limited to small numbers of animals but, nevertheless, provide preliminary proof that PrP polymorphisms can indeed affect PrP^CWD^ shedding and warrant further investigations with larger group sizes.

## 6. Transmission Barriers and Zoonotic Potential of CWD

With the emergence of CWD and its continuous spread, assessing the host range and transmission barriers to other species became a priority. A study by Bruce et al. (2000) demonstrated an inefficient transmission of PrP^CWD^ isolates to non-transgenic laboratory mouse strains [183], suggesting a strong transmission barrier of CWD. Later, studies with hamsters and transgenic mice expressing hamster PrP showed the susceptibility of these species to CWD at variable attack rate via intracerebral route [184]. Interestingly, CWD was reported to be efficiently transmitted to transgenic mice overexpressing murine PrP, while mouse-adapted PrP^CWD^ showed lympho-tropism and biochemical and histological characteristics that resembled those from CWD-infected cervids [185]. Moreover, emergence of different PrP^CWD^ strains, differing in disease incubation period and neuropathology, was observed when the mule deer PrP^CWD^ isolate was serially passaged into Syrian golden hamster (SGH) and transgenic mice expressing SGH hamster PrP [184]. North American wild rodents, such as meadow voles, deer mice, and white-footed mice, were susceptible to CWD infection via the intracerebral route and supported prion adaptation upon subsequent passaging [186]. Interestingly, transmission of CWD to bank voles resulted in very efficient transmission with 100% attack rate and short incubation period [187]. Besides, ferrets showed susceptibility to CWD challenge and were useful in dissecting PrP^CWD^ strains [188,189,190]. Additionally, CWD could transmit to cats, yet, with low attack rate and resulting in longer incubation periods. Attack rate increased, while incubation periods decreased, upon PrP^CWD^ adaptation in second passage [191]. Indeed, the feline-adapted PrP^CWD^ was able to transmit disease in cats following oral challenge which was not the case for cervid-derived PrP^CWD^ [191]. Notably, histological analysis revealed that disease produced by feline-adapted PrP^CWD^ in cats was distinct in terms of neuropathology when compared to that elicited by CWD and Feline Spongiform Encephalopathy (FSE), respectively [192]. A major challenge is the threat CWD poses to human health. CWD would have a significant impact on public health if it transmits directly to humans, or to potential intermediate hosts for transmission to humans, e.g., livestock [193,194,195]. When PrP^CWD^ from WTD was inoculated either i.c. or orally into pigs, PrP^Sc^ was detected in brain and lymphoid tissues even at 6 months post-inoculation by immunoassays and RT-QuIC, despite the absence of clinical disease [196]. Interestingly, the pig-adapted PrP^CWD^ had distinct electrophoretic mobility when compared to pig-adapted PrP^BSE^ and cervid PrP^CWD^ [196]. Besides, the PrP^CWD^-positive brain samples from some i.c. or orally PrP^CWD^-inoculated pigs produced prion disease in transgenic mice expressing porcine PrP [196]. Although the study in pigs showed low CWD transmission, the data suggest that the presence of CWD infectivity in pigs by bioassays are enough to raise serious concerns regarding the role of livestock and wild boar as intermediate hosts, either at the clinical or sub-clinical disease stage, in the zoonotic transmission of CWD [196]. Such cross-species CWD transmission could be critical in areas where deer and these intermediary hosts share pastures. Perhaps reassuringly, CWD transmission studies to cattle suggested limited susceptibility upon intracerebral challenge that result in an incomplete attack rate and unique disease feature, distinct from BSE, with no spongiform changes in the brain [197,198,199,200]. Recently, a 10-year follow-up study demonstrated the failure of CWD transmission to cattle either when they were orally challenged with mule deer PrP^CWD^ or co-housed with CWD-infected cervids and in PrP^CWD^-contaminated environment, suggesting a high transmission barrier of cattle to CWD upon natural exposure [201]. CWD transmission was also not successful to transgenic mice expressing heterologous PrP from different species, including bovine, ovine and human, indicating a high interspecies transmission barrier for CWD [202,203]. Of note, difference between the primary structure of the host PrP and invading PrP^Sc^ play a major role in species barrier [41,204,205,206]. Yet, more investigation into the potential of interspecies transmission of CWD is required, considering the facts that various PrP^CWD^ strains exist and that *Prnp* polymorphisms have an influence on strain generation, CWD pathogenesis and transmission [92,120,146]. Overall, the long incubation period, presence of different prion strains, effect of *Prnp* polymorphism, and atypical clinical presentation should be taken into consideration while assessing the threat posed by CWD.

The possibility of immediate transmission of CWD from cervids to humans has been a keen interest for prion researchers to address from the time when CWD was first detected. Moreover, the presence of PrP^CWD^ in the skeletal muscles and antler velvet is alarming due to meat consumption and the use of antler velvet in traditional Asian medicines [5,165]. Surveillance data from CWD-endemic areas suggested no link between the CWD prevalence and incidence of prion disease, either typical or atypical/novel, in humans [207]. There were also no differences in terms of human CJD prevalence in CWD endemic vs. non-endemic areas in Colorado from 1979 to 2001 [208]. Although a retrospective study in the U.S. reported that some people who developed CJD were exposed to CWD in their past through diet [209], in follow-up studies for six years no prion disease was reported for those exposed to CWD via consumption of PrP^CWD^-infected deer meat [210]. Yet, the long incubation period of prion disease should not be underestimated during such follow-up studies. In an in vitro study using PMCA, PrP^CWD^ could readily convert human PrP substrate into PrP^Sc^, though only after prion strain adaptation gained through subsequent passaging of PrP^CWD^ in PMCA or in transgenic mouse models [211]. Another study analyzed the efficiency of PrP^CWD^ to convert human PrP from the human and transgenic mouse brains; it found that human PrP was converted by PrP^CWD^ regardless of the polymorphism at codon 129 of human PrP, but M129 PrP was a more efficient substrate than V129 [36]. Interestingly, the migration and glycoform pattern of the proteinase K-resistant PrP^CWD^ -converted-human PrP was similar to that of MM1 sporadic CJD (sCJD) and distinct from vCJD [36]. Moreover, RT-QuIC assays revealed that both cervid PrP^CWD^ and feline-adapted PrP^CWD^ effectively seeded recombinant human PrP with seeding activity higher than PrP^BSE^ but was lower than sCJD [40]. In contrast to those in vitro findings, PrP^CWD^ inoculated i.c. into transgenic mice overexpressing human PrP showed no CWD transmission, suggesting a strong interspecies transmission barrier [202,203,212]. Recently, using RT-QuIC, PrP amyloid seeding activity was assessed in the brains of transgenic mice overexpressing human PrP after inoculating them with CWD prions from mule deer, WTD, and elk [213]. Although the mice failed to show any typical prion clinical disease, and PrP^CWD^ accumulation and neuropathology were not detected in the brains, RT-QuIC analysis detected amyloid seeding activity in the brains of a few PrP^CWD^-inoculated mice [213].

The zoonotic potential of CWD was further evaluated using non-human primates as an experimental model. Experimental CWD transmission was successfully achieved in squirrel monkeys by both intracranial and oral infection routes, but in contrast, not in cynomolgus macaques [214,215,216]. In a more recent transmission study of CWD to cynomolgus macaques performed by a Canadian-led consortium, CWD prions were able to transmit and cause clinical signs in some animals challenged i.c or orally, mimicking the natural transmission route [195,217]. These contradictory reports for CWD transmission to non-human primates indicate some zoonotic potential of CWD, especially keeping in mind of the emergence of novel natural CWD strains [32], the longer incubation periods that could be exerted by prions while transmitting to macaques [218], and the possibility of subclinical or atypical disease presentation. While solid evidence for CWD transmission to humans is lacking, the question whether CWD could cross the species barrier and transmit to humans is still a top priority and a serious public health concern. Another important question is how CWD would manifest in humans, whether it can be diagnosed with currently used assays, and whether it would further transmit among humans.

## 7. Conclusions

CWD is considered the most contagious prion disease. It is spreading efficiently among wild and farmed cervids, resulting in increasing case numbers and an expansion in geographic distribution. Principally, all cervid species that are currently exposed to CWD are highly susceptible to infection and clinical disease, but species-specific polymorphisms in *Prnp* on one or both alleles result in single amino acid substitutions in the PrP and modulate pathogenesis. In most cases, the presence of only one mutated allele extends the incubation period of infected animals. It is tempting to speculate whether breeding schemes selecting for animals with a genetic background of lower CWD susceptibility could be introduced as a management strategy to curb the spread of CWD, a practice that was successfully applied to manage scrapie in sheep. However, it is important to keep in mind that there is no absolute resistance to infection, and animals carrying less susceptible genotypes still propagate infectious prions and might shed them for a longer period of time during extended incubation. Datasets from experimental infection schemes of cervids carrying less susceptible genotypes addressing shedding are critically important but very limited, mostly due to the low number of animals with non-wild type genotypes in those studies. Another caveat is the emergence of a growing variety of PrP^CWD^ strains. Initially, it appeared that strain diversity in CWD is limited to basically two strains. In recent years, however, more PrP^CWD^ strains were isolated mostly from cervids expressing PrP with a polymorphism (e.g., H95^+^, 116AG, 132L), demonstrating another significant role of PrP^C^ primary structure variations in CWD pathogenesis. The main concern with these findings is that strains can have distinct host ranges, and for some of them clinical disease was achieved in a mouse model previously considered resistant to various PrP^CWD^ isolates. An expanded host range may not only cause increased transmission among cervids, but also transmission to sympatric species, livestock and humans. Recent evidence suggests some zoonotic potential of CWD, raising concerns as hunting, which is popular in North America and Scandinavia, and widespread consumption of venison exposes people to CWD.

It is clearly recognizable that cervid *Prnp* polymorphisms add an additional layer of complexity for management and risk assessment of CWD. Samples obtained for CWD surveillance can and should be utilized not only for CWD testing, but also for genotyping, strain typing, and transmission studies. Altogether, solving CWD-associated challenges calls for a One Health approach encompassing collaboration between molecular and wildlife biologists, social scientists, and regulators to eventually contain the spread and reduce the risks to ecosystem, animal, and human health.

## Figures and Tables

**Table 1:** Cervid prion protein polymorphisms reported to date.

Species	PrP Codon	Allele		Effect of Polymorphism on CWD Pathogenesis (In Vivo)	Geographic Location	Ref.
		wt	var			
**White-Tailed Deer (*Odocoileus virginianus*)**	95	Q	H	Prolonged survival, reduced susceptibility and reduced peripheral prion spread	U.S.: WI, NE CA: AB, SK	[102,103,104,105,106,107]
	96	G	S	Prolonged survival, reduced susceptibility, delayed lymphoreticular spread	U.S.: WI, NE CA: AB, SK	[13,102,103,104,106,107,108]
	116	A	G	Reduced susceptibility and lower infection rate	U.S.: NE CA: AB, SK	[101,106,109]
	226	Q	K	Reduced susceptibility and lower infection rate	U.S.: WI	[101,107,108]
	226	Q	R	N/A	CA: AB, SK	[109]
	230	Q	L	N/A	CA: AB, SK	[109]
**Mule Deer (*O. hemionus*)**	20	D	G	Possibly more susceptible	U.S.: WY, CO CA: AB, SK	[109,110]
	225	S	F	Slower disease progression	U.S.: WY, CO	[110,111]
**Elk (*Cervus canadensis*)**	132	M	L	ML and LL have increased disease incubation periods, LL has lesser grey matter and greater white matter spongiform change, less PrP^Sc^ accumulation, and more stable fibrils, and 132L alleles are twice as frequent in herds known to be infected for >30 years than uninfected herds	U.S.: CO, WY, other mid-western/western states (e.g., SD, ND, MT, NE, MI)	[112,113,114,115,116,117,118]
	226	E	-	More stable PrP^CWD^ strains, but less conformational stability.	CA U.S.	[119,120]
**Red Deer (*C. elaphus*)**	98	T	A	N/A	Britain, Czech Republic	[121]
	168	P	S	N/A	Britain	[121]
	226	Q	E	N/A	Britain, Norway, Czech Republic	[121]
	247	I	L	N/A	Czech Republic	[121]
**Sika Deer (*C. nippon*)**	226	Q	E	N/A	Britain	[121]
**Fallow Deer (*Dama dama*)**	138	N	-	Resistance to natural infection and prolonged incubation periods in intra-cerebrally infected animals	Britain	[122,123,124]
	226	E	-	N/A	Britain	[122,123,124]
**Reeve’s Muntjac Deer (*Muntiacus reevesii*)**	98	-	S	N/A	Britain	[121]
**Chinese Water Deer (*Hydropotes inermis inermis*)**	100	S	N	N/A	Britain	[121]
	OR *	5	4	N/A	Britain	[121]
**Korean Water Deer (*H. i. argyropus*)**	96	G	D	N/A	South Korea	[125]
	100	N	S	N/A	South Korea	[125,126]
	170	D	G	N/A	South Korea	[125]
**Moose (*Alces* sp.)**	36	T	N	N/A	Canada	[98]
	100	S	R	N/A	Canada	[127]
	109	K	Q	N/A	Sweden	[98]
	209	M	I	N/A	U.S.: WY, WK	[128,129]
**Caribou/Reindeer (*Rangifer tarandus* sp.)**	2	V	M	N/A	CA U.S.: AK	[130,131,132]
	129	G	S	N/A	CA U.S.: AK	[130,131,133]
	138	S	N	138SN and NN reindeer have prolonged incubation periods in oral transmission, and both also have no or limited PrP^CWD^ distribution in the CNS	CA: BC, YK, NT, AB, SK U.S.: AK	[130,131,133,134]
	153	Y	F	N/A	CA	[133]
	169	V	M	N/A	CA U.S.: AK	[130,131,133]
	176	N	D	N/A	Sweden, Norway CA: NWT	[98,133]
	225	S	Y	Higher risk of infection	Sweden, Norway	[98,135]
	242	P	L	N/A	CA	[133]
	OR	5	4	Higher risk of infection	Norway	[135]

* OR: octapeptide repeat.
